# Report of Five Cases of Empyema

**Published:** 1859-11

**Authors:** H. Noble

**Affiliations:** Heyworth, Ill.


ARTICLE IV.

### REPORT OF FIVE CASES OF EMPYEMA.

BY H. NOBLE, M.D., OF HEYWORTH, ILL.

*Mr. President and Gentlemen:*

At the time I was directed by the President to furnish and read a paper to the Society, I was treating a case of empyema, in which I was very much interested, and having my attention at that time drawn especially to that disease, I shall make it the subject of the following paper.

Empyema is a disease or consequence of disease of not very frequent occnrrence in this country. (I speak from observation only).

During a practice of fourteen years, I have seen five cases only; and from inquiry made by me of my friends, I learn that it is at least as seldom met with by the most of them as it has been with me.

Dr. Thompson, in his article on Empyema, in the *Cyclopedia of Practical Medicine*, gives a table furnished by Dr. Davies, containing ten cases, from which I infer that the number of cases occurring to those celebrated physicians were not numerous.

This, however, does not detract from the interest physicians should feel in a disease, every case of which is of vital importance to the patient. There is, perhaps, no condition of the system produced by disease more completely hopeless without efficient aid than the one under consideration. Although the books mention cases which were cured by absorption, we can only expect to meet such cases like angel visits—most welcome but most rare.

Empyema is a consequence or result of a very serious disease, namely, inflammation of the pleura. The occurrence of effusion is not evidence of the subsidence of the inflammation; although, so far as I have observed, the inflammatory action has in each case been materially modified by it, requiring the subsequent treatment to be directed not so much to the pleuritis as the effusion.

Notwithstanding the unfavorable condition of the system in which empyema occurs—namely, a pretty general inflammation of the pleural cavity—all the cases are not necessarily fatal, a fair proportion recovering after an operation. For instance, Dr. Davies, in his table, given by Dr. Thompson, reports ten operations, of which eight were successful. Dr. Bell says the term "successful" is too often used by surgeons to designate the operations that have been regularly and completely performed without the patient sinking under them. Of the applicability of that remark to Dr. Davies' cases I know nothing, but as to the cases which I shall report, you can judge for yourselves.

The first case I saw of empyema was that of John Munson, 10 years of age, who was attacked with pleuritis in the spring of 1838. The patient was not healthy, having been troubled with asthma from infancy. In the third week of his disease effusion took place in his left side. Immediately after the occurrence of the effusion, the inflammatory symptoms subsided; the pulse, which had been from 120 to 140, fell to 90 or 98, and the system required sustaining treatment. The side increased in size, and in a few days the intercostal spaces were bulged outward.

About the tenth day the heart pulsated on the right of the sternum, and by the fifteenth day there was a distinct pointing between the sixth and seventh ribs. An opening was made, and three half pints of pus discharged at once, after which there was discharged daily half a gill for two weeks. The discharge gradually diminished, and at the end of ten months entirely ceased and the opening closed. The side is considerably collapsed, but the lung admits air, but not in quantity equal to the other. The heart beats on the left side again, and the patient enjoys good health, except the asthmatical difficulty, which does not appear to have been modified by the disease.

The treatment after the operation was quinine, in moderate doses, muriated tincture of iron and iodide of potassium, mineral acids, etc. The diet was of the most nourishing kind.

The second case I saw was on the 1st of February, 1849, a son of Mr. Nickerson, 14 months old. Had effusion when I first saw it; the side, which was the right, being considerably enlarged, and unable to breathe unless the shoulders were elevated. The intercostal spaces protruded and the breast beat exterior to the left nipple.

I operated by making an opening between the sixth and seventh ribs, and evacuated two teacupfuls of pus. The relief was immediate, the little sufferer being asleep in thirty minutes.

This child was nursing at the time and took but little food for five or six days, after which it took new milk, soup, etc. Its medicine was nitric acid diluted and sweetened.

The discharge continued two months when it ceased, and the opening closed. The side is natural. The lung takes air equal to the other, and the boy is now large and healthy.

The third case I saw in 1849. The left side was greatly enlarged when I was called; breathing much labored; heart beating on right side of sternum. The father objected to an operation, and the boy died in two days. In this case the matter was admitted into the trachea, and was ejected in large quantities with great violence, some being thrown entirely across the room. Death occurred in a few minutes after the matter broke into the windpipe.

The fourth case was Mr. Scott, who, in 18—, was attacked with pneumonia, and received the ordinary treatment for that disease. He was freely bled early in his sickness.

About the twelfth day his side (the left) was discovered to be enlarged, and on the following day his heart beat on the right side of the sternum. On the third day after the enlargement was noticed, the dyspnœa was great, and a consultation was agreed upon for the next day, but the patient died before the time arrived.

This patient died from compression of the lungs, the accumulation being so great that respiration was rendered impracticable.

The fifth case was seen first on 22d November, 1858. The patient was attacked with pleurisy in the left side on the evening of the 21st, and I found him on the following morning suffering extreme pain inside, increased by breathing; cough troublesome, but difficult on account of the pain; pulse 120, full, but easily compressible. The bowels had been evacuated the previous day with sulph. mag. I gave calomel, ipecac., tartar and morphine, and applied sinapisms to the side.

Under this treatment he improved, and the third day I left him convalescent. In two days more I found him suffering with fever, little or no pain in his side, pulse 100 and small, tongue glossy, and tip and edges red. In short, he lay eight weeks with typhoid fever; after which, he got up and walked about the house, but did not get well. His pulse during this time was about 80, soft and full.

This condition lasted about four weeks, when he was taken with a chill, which was followed by fever and pain in left side. This pain was persistent, and was treated with blisters, nauseants and anodynes. Quinine was tried, but aggravated the symptoms.

In four weeks from the chill, the first symptoms of effusion were noticed, which was œdema of the left side and occlusion of the lower part of the left lung from the air.

In two days more the heart was observed to beat under the sternum, and in two days afterwards it beat on the right side of the sternum.

The air now scarcely penetrated the upper portion of the lung, and a careful measurement showed the *right* side to be nearly half an inch the largest, although the effusion was on the left. As the effusion increased the air was prevented from penetrating the lower part of the *right* lung, and the heart was crowded farther to the right side, until it beat under the outside edge of the right nipple, which was the farthest point of deflection from its natural position.

During the whole continuance of the disease the breathing was *not* labored nor hurried, but easy and almost natural. The right side also continued the largest.

I operated on Sunday, the 1st of May, 1859, and discharged a pint and a half of pus at two o'clock p.m., and again removed one pint at ten o'clock same night. One pint was discharged daily afterward, until the accumulation was removed, which amounted to six quarts by estimation, after which about one fourth of a pint was discharged daily for four or five weeks; then the discharge gradually diminished, and changed from pus to serum, and in about eight weeks from the operation ceased entirely and the opening healed.

The medical treatment this patient received was quinine, iodide of potassium, hypophosphite of lime and soda, and diet of the most nourishing kind.

The patient is now enjoying excellent health. The left lung is permeable to the air in its whole extent, and he bears exercise without any difficulty.

I attribute the complete success which attended this operation to two causes: First, to the opening in his side, which was *valvular* to such a degree that the pus would not flow through it at any time, having to pass a tube every time the matter was removed. This prevented the air from entering the pleural cavity except when the tube was used, at which time air always passed both in and out of the side. Now, my own opinion is this, that the air should be as much as possible excluded from the pleural cavity, because it adds to the irritation and consequently to the danger of the patient, and necessarily prolongs the discharge; but that it increases the danger to the extent generally taught I do not think. The first and second cases both admitted the air freely, and after a few weeks the air passed freely in first case at all times, if the position of the person enlarged or compressed the chest, notwithstanding which the the case terminated favorably, although not so soon as the fifth case.

The second cause to which I attribute the good success of the fifth case, and it bears equally on all the cases operated on, is the system of diet. No pains were spared to nourish the patient with food, which was given in considerable variety, no meal being made of one course. Pure wine from the native grape was given liberally, and new milk was also taken regularly and freely.

To sum up, I would recommend that the opening be made so valvular, if possible, that the accumulated fluid would not escape without the insertion of a tube. That the fluid be discharged a little at a time, never withdrawing more than enough to remove all tension at the first operation. That the vitality of the patient be sustained by all possible means, especially by nutritious diet.

By this means I have succeeded in restoring the cases reported without their assuming a chronic form, which would require local applications to stimulate and charge the secretory action of the pleura.

Should I meet such a case, I should not fail to follow the example and direction of Dr. Brainard, to inject the pleural cavity with iodine. This, to say the least, appears like a heroic operation; but after his reasoning and experience, I should not hesitate to resort to it in any case in which it was not contraindicated by lesions of structure which would render the case hopeless independent of the pleura effusion.

NOTE BY THE EDITOR.

Our thanks are due to the Clinton Co. Society for the above valuable paper. In the case of empyema, treated by injections of iodine, as reported by us in a former number of this Journal, an error occurred which it is important to correct. The case was of five years instead of five months' standing. We have now under treatment at the Chicago Hospital, a case of empyema of ten months' standing. It was the result of a stab. We throw a solution of one grain of iodine, with thrice the quantity of iodide of potassium to the ounce of distilled water, into the opening twice a week.

This method of treatment is not new or peculiar to ourselves, but has been used successfully by Boinet and others in France several years since.

